# Emerging role of γδ T cells in vaccine‐mediated protection from infectious diseases

**DOI:** 10.1002/cti2.1072

**Published:** 2019-08-28

**Authors:** Kathleen W Dantzler, Lauren de la Parte, Prasanna Jagannathan

**Affiliations:** ^1^ Department of Medicine Stanford University Stanford CA USA

**Keywords:** cytokines, infection, proliferation, vaccination, Vγ9Vδ2 T cells, γδ T cells

## Abstract

γδ T cells are fascinating cells that bridge the innate and adaptive immune systems. They have long been known to proliferate rapidly following infection; however, the identity of the specific γδ T cell subsets proliferating and the role of this expansion in protection from disease have only been explored more recently. Several recent studies have investigated γδ T‐cell responses to vaccines targeting infections such as *Mycobacterium*,* Plasmodium* and influenza, and studies in animal models have provided further insight into the association of these responses with improved clinical outcomes. In this review, we examine the evidence for a role for γδ T cells in vaccine‐induced protection against various bacterial, protozoan and viral infections. We further discuss results suggesting potential mechanisms for protection, including cytokine‐mediated direct and indirect killing of infected cells, and highlight remaining open questions in the field. Finally, building on current efforts to integrate strategies targeting γδ T cells into immunotherapies for cancer, we discuss potential approaches to improve vaccines for infectious diseases by inducing γδ T‐cell activation and cytotoxicity.

## Introduction

Although representing only a small percentage of T cells (generally 2–5% of peripheral blood T cells in healthy adults), γδ T cells have increasingly been recognised for their unique roles in establishing and regulating the inflammatory response to infectious diseases. These unconventional T cells have antigen recognition capacity, tissue tropism and cytotoxic functions that are distinct from αβ T cells. γδ T cells are the first T cells to appear in the thymus during foetal thymic ontogeny and, following gene rearrangement, express different T‐cell receptor (TCR) sequences.^1^ TCR diversity is different across different animals, but in humans, subsets expressing different Vγ and Vδ regions localise to different tissues and have differing effector functions. For example, the most abundant subset in human adult peripheral blood is Vγ9Vδ2 cells (also referred to as Vγ2Vδ2) while Vδ1^+^ cells are more common in mucosal tissues.^2^ Existing only in primates, Vγ9Vδ2 cells recognise phosphoantigens induced by stress or pathogens in a process that is dependent on butyrophilin 3A1 (BTN3A1, CD277), a type I glycoprotein in the B7 family.^3^ Other signalling pathways for human γδ T‐cell activation involve TCR interaction with ligands such as F1‐ATPase or endothelial protein C receptor, or additional cell surface receptors such as natural killer group 2 member D (NKG2D) receptors or toll‐like receptors (TLR).^4^ Unlike αβ T cells, all of these pathways are independent of the major histocompatibility complex (MHC). In some animals (e.g. cattle, sheep, chickens), γδ T cells express highly diverse TCRs regardless of tissue localisation, while in others (e.g. mice), almost all γδ T cells in the epidermal layer of the skin (called ‘dendritic epidermal T cells’) express identical γδ TCRs. Interestingly, γδ TCRs are structurally more similar to immunoglobulins than αβ TCRs; the CDR3 lengths of TCR δ chains are long and variable, whereas those of the TCR γ chains are short and constrained.^1^ The presence of TCR chains that use antibody‐like V domains is widely distributed in vertebrates, suggesting a selective pressure for TCR chains that recognise antigen in ways similar to that of antibodies.

Several γδ T‐cell subsets have long been known to rapidly increase in number following systemic infections and to perform numerous roles, including direct anti‐microbial roles, recruitment of innate immune cells and activation of adaptive immune cells.^4^ In many situations, including most bacterial and parasitic infections in humans, it is the Vδ2^+^ T‐cell subset that proliferates, while in some viral infections, Vδ1^+^ T cells expand and exert anti‐microbial activities. Interestingly, γδ T cells also appear to have some level of functional plasticity, enabling them to adapt their function at different points during infection based on TCR signalling and environmental cues. Animal models have further provided support that these cells are not simply biomarkers of infection, but can in fact mediate protection from disease and/or recurrent infection. Despite being known to have an important role in immunity to infectious diseases, γδ T cells have, with the exception of the Bacillus Calmette–Guérin (BCG) vaccine for tuberculosis, largely been ignored in vaccine development. Whether γδ T cells are stimulated directly by the antigen component of the vaccine or indirectly with an appropriate adjuvant, there may be many opportunities to improve vaccine effectiveness by targeting γδ T cells. In this article, we will review the evidence for the role of γδ T cells in vaccine‐induced protection to bacterial, protozoan and viral infections. Many of these diseases, particularly those responsible for the highest mortality and morbidity worldwide – tuberculosis, malaria and HIV – do not yet have an effective vaccine because of rapid pathogen evolution and other biological and technical challenges. However, considering the functional roles of γδ T cells and incorporating them into a vaccine strategy could be an important step towards reducing the devastating impact of these diseases.

## Mycobacteria and other bacterial infections

A number of studies have shown expansion of γδ T‐cell populations in response to various bacterial infections, both in humans and in animal models. In humans, γδ T cells accumulate at mucosal epithelial tissues, including the lungs,^5^ and have been shown to rapidly proliferate following infection with *Mycobacterium tuberculosis (Mtb)*.^6^, ^7^ These responding γδ T cells primarily express Vγ9Vδ2^8^ and recognise *Mtb* phosphoantigen.^6^, ^9^ Studies testing whether γδ T cells expand in response to the *Mtb* heat shock protein HSP65 have had somewhat conflicting results, but suggest that while some γδ T‐cell clones can recognise HSP65, the majority of cells respond to other antigens.^7^, ^10^, ^11^ Several *in vitro* studies have suggested that Vγ9Vδ2 T cells may mediate protection from *Mtb*. These cells appear to be capable of directly killing extracellular *Mtb* via release of granulysin and intracellular *Mtb* via granulysin and perforin.^12^ Mycobacteria‐specific Vγ9Vδ2 T cells from individuals positive for the tuberculosis skin test also produce granzyme A, which indirectly leads to *Mtb* destruction by stimulating TNFα production by infected macrophages.^13^ In the mouse model, although γδ T cells seem to be less essential to immunity against *Mtb*,^14^, ^15^ GM‐CSF production by γδ T cells in the lungs seems to play a role in protection and an additive effect between GM‐CSF and IFNγ promoted macrophage control of intracellular bacterial replication.^16^ Clearly, the Vγ9Vδ2 T‐cell subset is important in the human immune response to *Mtb*, but further work is required to evaluate the role of various cytokines in protection from disease at different timepoints during infection.

γδ T cells also seem to play a role in immunity induced by BCG, the only current vaccination against *Mtb*. Similarly to natural infection, γδ T‐cell populations expand and produce IFNγ in response to BCG vaccination.^17^, ^18^, ^19^ In fact, IFNγ production by these cells was greater than that of CD4^+^ T cells.^19^ In adults, Vδ2^+^ γδ T cells from BCG‐vaccinated individuals expanded more than cells from non‐vaccinated individuals in response to *in vitro Mtb* restimulation; this memory‐like phenotype could not solely be attributed to increased helper functions from mycobacteria‐specific memory CD4^+^ T cells.^20^ Given that BCG contains lower levels of phosphorylated nonpeptidic antigens compared to *Mtb*,^21^ it is unclear whether γδ T cells responding to BCG are recognising the same or different antigens compared to natural infection. Further studies are needed to evaluate the functional role of γδ T‐cell expansion following BCG vaccination, including any role for memory‐like subsets and whether expansion provides protection upon challenge or infection with *Mtb*. Considering the importance of granulysin, perforin and granzyme A in response to *Mtb*, it may also be useful to incorporate strategies that elicit these responses into vaccine design.

Studies in non‐human primates further support an important role for γδ T cells in responding to *Mtb* infection and BCG vaccination. These studies may additionally provide insight into mechanisms driving immunity induced by γδ T‐cell expansion. Non‐human primates serve as a useful model as they also express the Vγ9Vδ2 T‐cell subset, which recognise *Mtb*, unlike murine γδ T cells which do not recognise phosphoantigen or microbial antigens.^15^ Administration of an *Mtb* phosphoantigen analog combined with IL‐2 expanded the Vγ9Vδ2 T‐cell population during *Mtb* infection.^22^ Expanded Vγ9Vδ2 T cells differentiated into effector subpopulations, expressed cytokines such as IFNγ, perforin, granulysin and IL‐12, and led to enhanced pulmonary responses of peptide‐specific CD4^+^/CD8^+^ T cells.^22^ Importantly, diminished TB lesions and reduced *Mtb* proliferation were also observed, suggesting a role for expanded/differentiated Vγ9Vδ2 T cells in resistance to *Mtb* infection.^22^ In another approach, adoptive transfer of autologous Vγ9Vδ2 T cells 1 or 3 weeks after *Mtb* infection led to significant protection from *Mtb*, including a rapid recall‐like increase in the pulmonary Vγ9Vδ2 T‐cell subset, decreased *Mtb* infectious burdens (particularly in the lungs) and reduced pathology.^23^ Following BCG vaccination, Vγ9Vδ2 T cells expanded as early as 4–6 days post‐vaccination with peak levels at 3–5 weeks post‐vaccination; this expansion further coincided with clearance of bacteraemia and immunity to fatal tuberculosis after challenge.^24^ Finally, a prime‐boost approach using phosphoantigen followed by fusion proteins led to expansion of γδ T cells displaying effector memory surface markers and producing cytokines such as IL‐2, IL‐6, IFNγ and TNFα following primary vaccination.^25^ As these cells anergised following boosts whereas αβ T cells expanded,^25^ future studies could investigate whether anergy can be prevented and γδ T‐cell recall responses preserved. Together, the described studies in macaques provide evidence that γδ T cells confer protection from symptomatic *Mtb* infection and support targeting these cells in vaccination approaches to *Mtb*.

The γδ T‐cell ontogeny is quite different in other mammals compared to humans and non‐human primates; however, studies in cattle and pigs showed similar responses to those found in humans and macaques. Cattle and other ruminants express large proportions of γδ T cells that decline with age, but remain high relative to human levels.^26^, ^27^ In cattle, γδ T cells rapidly proliferate following infection with *Mycobacterium bovis* 28, 29, 30 or BCG vaccination.^31^, ^32^ Similarly, in pigs, γδ T cells proliferated following vaccination with BCG.^33^

Other bacterial agents demonstrating γδ T‐cell expansion following infection and vaccination include *Leptospira borgpetersenii*,* Salmonella enterica*,* Francisella tularensis* and *Listeria monocytogenes*. Similarly to the described response to *Mtb*, human γδ T‐cell populations, in particular the Vγ9Vδ2 subset, expand following leptospirosis infection.^34^, ^35^ In leptospirosis vaccination studies in cattle, IFNγ‐producing γδ T cells expressing the WC1 co‐receptor expand post‐vaccination and upon *in vitro* restimulation.^36^, ^37^, ^38^ γδ T cells also expand following salmonella vaccination in chickens and macaques^39^, ^40^ or following salmonella infection in humans.^41^ Furthermore, following salmonella or listeria vaccination in macaques, γδ T cells displaying Vγ9Vδ2 were the major T‐cell subset proliferating.^40^, ^42^ Following subclinical *Listeria monocytogenes* infection, Vγ9Vδ2 T cells expanded, trafficked to the lungs and intestinal mucosa and evolved into effector cells producing IFNγ, TNFα, Il‐4, Il‐17 and/or perforin.^42^ These cells could then lyse infected target cells and inhibit intracellular bacterial growth, demonstrating a potential role in protection from listeria.^42^ Interestingly, γδ T cells displaying Vγ9Vδ2 expanded in humans infected with *F. tularensis,* 43, 44 but did not expand following vaccination, perhaps because of different phosphoantigens present.^43^

In summary, a number of studies have not only demonstrated γδ T‐cell expansion in various bacterial infections, but also possible mechanisms of protection provided by this cell population, including both direct killing and recruitment of other cell types via production of pro‐inflammatory cytokines. Although clear that γδ T cells respond differently based on infectious agent, specific proliferation of the Vγ9Vδ2 subset in response to a number of bacterial pathogens correlates with protection from symptomatic disease. Consequently, upregulating activation and/or functional responses of this subset by vaccination may enhance protection against the agent targeted by immunisation. However, given the γδ T‐cell anergy observed in the described vaccine study combining phosphoantigen with a subunit anti‐tuberculosis vaccine,^25^ as well as prevalent examples of T‐cell exhaustion in other contexts, further work is needed to assess potential mechanisms driving such processes. Timing of interventions could therefore be optimised to induce maximal γδ T‐cell recall responses and promote activation without causing exhaustion.

## Malaria infection

In addition to long‐standing evidence that γδ T cells play a role in initial responses to parasitic infections, there is increasing evidence that γδ T cells are important in vaccine‐induced protection from malaria. Studies over the past few decades have shown that γδ T cells (particularly the Vδ2^+^ subset) rapidly expand following infection with the most virulent human malaria parasite, *Plasmodium falciparum (Pf)*, in children, malaria‐naïve adults and malaria‐experienced adults.^45^, ^46^, ^47^, ^48^ Frequencies of γδ T‐cell subsets, including Vδ2^+^, Vδ2^−^, activated CD11c^+^ or CD16^+^/Tim‐3^+^ γδ T cells, have all been associated with malaria exposure.^49^, ^50^, ^51^, ^52^, ^53^, ^54^, ^55^, ^56^ Higher frequencies and malaria‐responsive cytokine production of Vδ2^+^ T cells correlate with protection against subsequent infection in children living in endemic settings,^57^, ^58^ and *in vitro*, these cells perform cytotoxic, anti‐parasitic functions.^59^, ^60^ Furthermore, these cells can also act as antigen‐presenting cells,^61^, ^62^, ^63^, ^64^ which may further enhance the response to infection and/or vaccination. In malaria‐naïve volunteers exposed to *Pf*‐infected mosquitoes, while under chloroquine prophylaxis, γδ T cells expand after infection.^65^ Elevated frequencies of γδ T cells expressing effector memory surface markers and enhanced responsiveness to *Pf* stimulation persist for over 1 year following experimental infectious challenge.^65^ A recent small study from the same group reported that vaccination with BCG changed the course of experimental malaria infection and that BCG vaccination was associated with altered innate immune activation (including γδ, NK and monocytes) following malaria challenge. Interestingly, expression of the activation marker CD69 on both NK cells and γδ T cells was associated with reduced parasitaemia.^66^ Trends towards increased degranulation and granzyme B production among γδ T cells from BCG‐vaccinated volunteers compared to unvaccinated were also observed.^66^ Together, these results suggest an important role for γδ T cells in mediating protective immunity to malaria.

Although there is not yet an effective vaccine for malaria, preliminary studies testing whole parasite vaccines in humans and mice suggest an important role for γδ T cells in protection from subsequent infection. The malaria vaccine that has advanced farthest to date is the RTS,S vaccine, which is based on the *Pf* circumsporozoite (CSP) protein and targets the sporozoite and liver stages of infection. Interestingly, RTS,S phase 3 trials in African children detected no significant change in γδ T‐cell frequencies following vaccination and minimal cytokine production by these cells in response to *in vitro* CSP stimulation.^67^ However, as the authors examined total γδ T cells rather than Vδ2^+^ or other γδ T‐cell subsets, it will be important for future studies to determine whether specific subsets correlate with protection and if so, whether future RTS,S formulations can target these subsets. RTS,S trials in malaria‐naïve populations have generally focused on anti‐CSP antibody studies and CD4^+^/CD8^+^ T‐cell responses without examining innate populations like γδ T cells. One recent study utilising a systems approach identified natural killer (NK) cell signatures that correlated with and predicted protection,^68^ suggesting that depending on the precise vaccine regimen, innate immune responses could be significant.

In contrast to RTS,S, vaccine formulations using sporozoites (the stage of the parasite injected by the mosquito into the human) have indicated a direct or indirect role for γδ T cells in protection. In malaria‐naïve individuals immunised with the attenuated *Pf* sporozoite (PfSPZ) vaccine, Vδ2^+^ T cells expanded in a dose‐dependent fashion and frequencies of these cells correlated with protection more significantly than any other cellular immune responses.^69^, ^70^, ^71^ Numbers of memory Vδ2^+^ T cells also correlated with protection in a recent PfSPZ trial in a malaria‐endemic region in Mali.^72^ Finally, when malaria‐naïve individuals were immunised with non‐irradiated PfSPZ combined with chemoprophylaxis (PfSPZ‐cVAC), the frequency of Vδ2^+^ T cells increased in a dose‐dependent manner and memory γδ T cells specifically increased expression of IFNγ and the activation marker CD38.^73^ Additional work is needed to further elucidate the mechanism of Vδ2^+^ T‐cell‐induced protection, as well as to determine whether frequencies of these cells could be used as a biomarker for protection in PfSPZ vaccinations in malaria‐endemic regions.

In the mouse model, results have depended somewhat on the parasite strain used, but generally support γδ T cells as a correlate of natural and vaccine‐induced protection. In the lethal *Plasmodium berghei* ANKA model, γδ T cells were not required to prevent infection upon blood‐stage challenge following sporozoite vaccination, but did contribute to pre‐erythrocytic immunity by recruiting dendritic cells and CD8^+^ T cells.^72^ These cells may also be important in modulating functional T follicular helper (Tfh) cell and germinal centre B‐cell responses.^74^ In contrast to these indirect roles in protection, γδ T cells appear to act as important effector cells following vaccination with nonlethal *Plasmodium yoelii* sporozoites.^75^ Results from mice lacking αβ T cells further suggest that γδ T‐cell cytotoxicity may become more effective after interaction with CD4^+^ T cells.^75^ Mice lacking γδ T cells further reveal that these cells may be particularly important in immunity targeting the liver stages of the parasite (before it enters the bloodstream).^76^ Clearly, it will be important to evaluate whether these differing results between murine parasite strains are solely because of differences in the type of immunity induced (i.e. *P. berghei*‐irradiated sporozoite vaccination induces sterile immunity, while *P. yoelii* vaccination does not). Interestingly, a vaccine using whole lysate of the promastigote stage of a related parasite, *Leishmania amazonensis*, led to protection against subsequent infection that was dependent on the presence of γδ T cells.^77^ The mechanisms driving this protection and implications for malaria vaccines, however, are unknown.

In sum, results from vaccination studies targeting malaria (and potentially other parasitic infections such as leishmaniasis) strongly suggest that γδ T cells play an important role in protection from future infection. However, future work is required to definitively show that γδ T cells directly mediate protection rather than act as a biomarker of infection, as well as to determine the mechanism of protection and the role of Vδ2^−^ subsets (if any). In particular, it will be important to assess whether protection is mediated via direct γδ T‐cell cytotoxicity and/or more indirect effects such as antigen presentation, recruitment of other cell types, or stimulation of functional Tfh cells and antibodies. Given that most malaria vaccines in trials, including the leading RTS,S vaccine, use specific antigens rather than whole sporozoites, vaccine effectiveness may be improved by the addition of an adjuvant or other vaccine component that stimulates γδ T‐cell responses. BCG vaccination may be a potential approach based on recent results of increased activation of innate cell populations following CHMI in BCG‐vaccinated individuals;^66^ however, given that this response only occurred in half of the vaccinated volunteers and the sample size was small, further study is warranted.

## Viral infections

There is evidence that γδ T cells may play a role in response to viral infections, including influenza virus, HIV and cytomegalovirus (CMV), and that they can directly kill virally infected cells. There is also evidence that these cells can expand *in vivo* in response to bisphosphonate stimulation and viral vaccination strategies and may contribute to improved outcomes, thereby raising the possibility that these cells could be targeted to play an important role in vaccine‐mediated protection.

Regarding influenza, several studies have shown that phosphoantigen or pamidronate‐activated γδ T cells are capable of inhibiting virus replication by killing influenza‐infected macrophages^78^ and/or lung alveolar epithelial cells.^79^ Phosphoantigen‐activated cells also have non‐cytolytic activities in response to pandemic H1N1, producing IFNγ and expressing inflammatory chemokines.^80^ Relatedly, it was also recently shown that Vγ9Vδ2 T cells can promote CD4^+^ T follicular helper cell differentiation, B‐cell class switching and influenza virus‐specific antibody production in an *in vitro* co‐culture assay,^81^ suggesting that these cells may provide both a direct cytotoxic and potential synergistic role in the adaptive immune response to influenza.

Although both inactivated and live attenuated influenza vaccine reduce influenza illness and disease complications, live attenuated influenza vaccine has been shown to have superior efficacy in children.^82^ Influenza‐responsive γδ T cells were found to expand following live attenuated, but not inactivated, influenza vaccination,^83^, ^84^ suggesting a potential immunologic correlate for this observation. Despite not proliferating after vaccination, γδ T cells in elderly individuals receiving the inactivated vaccine did increase perforin production and, after *in vitro* restimulation, proliferated and produced IFNγ and IL‐4.^84^ Similarly, the γδ T‐cell response in the nasal mucosa was attenuated in cigarette smokers relative to non‐smokers,^85^ suggesting these cells may represent a correlate for why smokers respond less well to influenza vaccination. In a murine model of influenza, γδ T cells significantly expand in bronchial alveolar fluid following infection,^86^ and in a humanised mouse model, pamidronate administration to mice reconstituted with human PBMC reduced disease severity and mortality following H1N1 and H5N1 influenza infection. However, pamidronate had no effect in mice reconstituted with Vδ2^−^depleted cells.^87^ Together, these studies suggest that γδ T cells may not only represent an immunologic correlate of protection from influenza infection and vaccination, but that they might also be a mediator of protection.

Regarding HIV, it has long been known that both the Vδ1^+^ and Vδ2^+^ subsets of γδ T cells have cytotoxic capacity against HIV^88^, ^89^, ^90^ and can inhibit viral replication *in vitro*. HIV‐infected elite controllers have elevated levels of Vδ2^+^ T cells compared with HIV‐negative controls or HIV‐infected individuals on antiretroviral therapy,^91^, ^92^ suggesting a potential role for these cells in inhibiting viral replication *in vivo*. γδ T cells may also play a role in controlling viral infection at mucosal barriers. A recent study reported that higher levels of pro‐inflammatory Vδ1^+^ T cells correlated with lower gut‐associated HIV viral load,^93^ and another study in rhesus macaques found that levels of CD8^+^ Vδ2^+^ T cells in the female reproductive tract correlated with lower SIV viral loads.^94^ Vδ1^+^ T cells expanding in HIV‐infected individuals may also protect from other infections. For example, Vδ1^+^ T cells producing IFNγ and IL‐17A responded to *Candida albicans* 95 and further expanded upon influenza vaccination combined with the MF59 adjuvant.^96^

Individuals with chronic HIV infection have been found to have Vδ2^+^ T‐cell depletion and dysfunction in response to phosphoantigenic stimulation.^97^ It is possible, however, that some of these cells are not dysfunctional but rather have different functions. For example, He *et al*. identified a population of CD16^+^ Vδ2^+^ T cells that had decreased responses to phosphoantigens but increased capacity for antibody‐dependent cellular cytotoxicity (ADCC). A decline in this population was associated with faster disease progression, while no decline was observed in individuals with controlled infection.^98^ Administration of zoledronic acid with IL‐2 in HIV‐infected, antiretroviral naïve patients was associated with Vδ2^+^ T‐cell expansion, dendritic cell activation and increased HIV‐specific CD8^+^ T‐cell responses.^99^ It was also recently shown that γδ T cells can be isolated from antiretroviral suppressed, HIV‐infected individuals and that these cells can kill autologous HIV‐infected CD4^+^ T cells. In addition, these cells could expand *ex vivo* following pamidronate stimulation and could significantly reduce viral replication, suggesting a potential role for these cells to clear HIV infection from latent reservoirs.^100^

Even though HIV vaccine trials to date have not investigated any changes in γδ T‐cell populations, an intriguing study looked at canarypox as a vector for HIV antigens and, after *in vitro* expansion, identified a Vγ9^+^ population (specific for canarypox, not HIV antigens) that produced IFNγ.^101^ These results suggest that in addition to adjuvants, vaccine vectors could be used to target γδ T‐cell responses.

Finally, in the context of CMV infection, oligoclonal γδ (primarily Vδ2^−^) T cells expand and differentiate into effector/memory cells.^102^, ^103^, ^104^, ^105^ Expansion of Vδ2^−^ T cells is associated with viral clearance both in immunosuppressed^102^, ^106^, ^107^ and in healthy populations.^102^, ^107^ These cells likely contribute to viral clearance via effector functions such as cytotoxicity and IFNγ/TNFα production,^108^ ‘antibody‐dependent cell‐mediated inhibition’,^109^ and enhanced cytotoxicity via sensing of IL‐18 from virus‐infected cells.^110^ During secondary infection, cells proliferate and resolve infection faster, suggesting a memory‐like phenotype.^102^ Several studies in mice have shown that (1) γδ T cells are capable of protecting αβ T‐cell‐deficient mice against CMV‐induced pathology and (2) adoptive transfer of CMV‐induced γδ T cells provides long‐term protection in immunodeficient mice.^111^, ^112^ These results suggest that γδ T cells are important mediators of protection against CMV and support approaches using adoptive transfer of effector/memory γδ T cells or targeting γδ T cells in future CMV vaccine trials. The possibility of inducing exhausted γδ T cells would need to be considered, however, as CMV infection has both been shown to result in higher numbers of these cells.^113^

In sum, results from *in vitro* and natural infection studies suggest an important role for γδ T cells in controlling influenza, HIV and CMV viral replication. Targeting γδ T cells through stimulation could provide an important adjuvant‐type role in vaccination and/or cure‐related strategies for viral infections.

## Conclusions

Across the different bacterial, protozoan and viral infections examined (summarised in Table 1), there are clear patterns of γδ T‐cell expansion, particularly of the Vδ2^+^ subset, in response to both infection and vaccination. In several contexts, including infection with *Mtb*, malaria, influenza and HIV and vaccination with BCG, PfSPZ and live attenuated influenza, γδ T cells are associated with protection. Further, evidence so far supports a role for γδ T cells in mediating protection via direct killing and other mechanisms. Studies in animal models, such as BCG vaccination in macaques and PfSPZ vaccination in mice, are beginning to shed light on direct mechanisms of protection *vs*. stimulation of other immune cells that mediate protection. Clearly, future work is needed to further elucidate these mechanisms, as well as the host and infection‐mediated factors that influence responsivity of γδ T cells and the relevant differences between responses to natural infection compared to response to vaccination. As new vaccine formulations targeting these diseases progress through development, the question of whether to induce γδ T cells or γδ T‐cell subsets will become an important consideration. In fact, this approach is already being implemented in cancer, whether via administration of Vγ9Vδ2 T‐cell agonists^114^ or using BCG to stimulate Vγ9Vδ2 T cells as treatment for bladder cancer.^115^, ^116^ Approaches incorporating γδ T cells into strategies targeting B‐ or T‐cell responses have also been promising so far. For example, as previously mentioned, a study testing a subunit tuberculosis vaccine combined with phosphoantigen observed a robust γδ T‐cell response, including expression of effector memory markers, following primary vaccination.^25^ Finally, another intriguing approach is to expand functional γδ T cells *ex vivo*, as has been tested with effector cells capable of inhibiting HIV replication^100^ and *Mtb* infection.^23^

**Table 1:** Human γδ T‐cell responses to bacterial, viral and protozoan infections and corresponding vaccinations

Author, year	Agent	Cohort	γδ T‐cell subset	Impact of infection/vaccination on γδ T‐cell activation	Associations between γδ T‐cell features and function/clinical outcomes
Bacterial					
Barnes *et al*. 1992^6^	*Mycobacterium tuberculosis (Mtb)*	Adults with tuberculous infection	All γδ	Strong correlation between expansion of γδ T cells and *Mtb*	*Mtb*‐reactive γδ T cells produced IFNγ, GM‐CSF, IL‐3 and TNFα; secretion of macrophage‐activating cytokines may contribute to resistance against mycobacterial infection
Dieli *et al*. 2001^12^	*Mtb*	PPD‐positive adults	Vγ9Vδ2		Vγ9Vδ2 T lymphocytes efficiently kill extracellular and intracellular *Mtb* through release of granulysin and perforin
Spencer *et al*. 2013^13^	*Mtb*	PPD‐positive, HIV‐negative adults	Vγ9Vδ2		Infected macrophages co‐cultured with γδ T cells produced TNFα and inhibited intracellular mycobacterial growth
Hoft *et al*. 1998^20^	Bacille Calmette–Guérin (BCG)	Adults	All γδ	γδ T‐cell expansion after vaccination; memory‐like immune responses after *in vitro* restimulation	Enhanced responsiveness after BCG vaccination suggests that γδ T cells are important to secondary immune response
Mazzola *et al*. 2007^17^	BCG	Infants	All γδ	Remarkable expansion of γδ T cells in response to vaccination	
Tastan *et al*. 2005^18^	BCG	Infants	All γδ	Significant increase in γδ T cells following vaccination at birth	
Zufferey *et al*. 2013^19^	BCG	Adults, children and infants	All γδ/Vδ2^+^	γδ T cells (particularly Vδ2^+^ subset) from infants and children immunised with BCG expand after *in vitro* restimulation	Vδ2^+^ T cells produce IFNγ following BCG vaccination
Barry *et al*. 2006^34^	Unknown *Leptospira* species	Adult case study	All γδ	Patient had an almost tenfold increase of γδ T cells above baseline following infection	γδ T‐cell expansion parallels patient's symptoms; unable to determine whether γδ T cells play role in resolution of or exacerbation of symptomatic disease
Klimpel *et al*. 2003^35^	*Leptospira interrogans*	Adults	All γδ	Preferential *in vitro* expansion of TCR+ γδ T cells in cultures exposed to high numbers of *Leptospira*	
Workalemahu *et al*. 2014^40^	lytB‐ aroA‐ *Salmonella enterica* serovar Typhimurium SL7207	Adults	Vγ9Vδ2	LytB negative vaccines stimulated large *ex vivo* expansions of Vγ9Vδ2 T cells from human donors	
Poquet *et al*. 1998^43^	*Francisella tularensis* and *F. tularensis* live vaccine strain (LVS)	Adults	Vγ9Vδ2	Massive increase in Vγ9Vδ2 T cells during infection; minor or no increase in Vγ9Vδ2 T cells after live strain vaccination	
Protozoan					
Ho *et al*. 1990^45^	*Plasmodium falciparum (Pf)*	Individuals (age not reported) with acute infection	All γδ	γδ T cells expand after infection and remain elevated for at least 4 weeks	
Roussilhon *et al*. 1994^48^	*Pf*	Malaria‐naïve adults with acute infection	All γδ	γδ T cells expand and remain elevated for months; subset proliferates *in vitro* in response to *Pf* schizont extract	
Hviid *et al*. 2001^46^	*Pf*	Children with acute infection	Vδ1^+^	Vδ1^+^ T cells increase after treatment	Expanded Vδ1^+^ T cells produce pro‐inflammatory cytokines
D'Ombrain *et al*. 2008^57^	*Pf*	Children in malaria‐endemic region	All γδ		Production of IFNγ following *in vitro Pf* stimulation associated with immunity to symptomatic infection
Cairo *et al*. 2014^51^	*Pf*	Neonates in malaria‐endemic region	Vδ2^+^	Neonates exposed to placental malaria had increased proportions of central memory Vγ2Vδ2 cells in cord blood	
Jagannathan *et al*. 2014^54^	*Pf*	Children in malaria‐endemic region	Vδ2^+^	Repeated infection associated with loss and dysfunction of Vδ2^+^ cells, including increased expression of immunoregulatory genes (Tim3, CD57, CD16)	Loss and dysfunction of pro‐inflammatory Vδ2^+^ cells associated with clinical tolerance to infection
Farrington *et al*. 2016^52^	*Pf*	Children in malaria‐endemic region	Vδ2^+^	High prior malaria exposure leads to increased CD16 expression on Vδ2^+^ T cells	High prior malaria exposure leads to lower Vδ2^+^ T‐cell functional responses; antimalarial chemoprevention associated with enhanced Vδ2^+^ cytokine production
Jagannathan *et al*. 2017^58^	*Pf*	Children in malaria‐endemic region	Vδ2^+^	Repeated infection associated with loss and dysfunction of Vδ2^+^ cells, including reduced proliferation	Higher pro‐inflammatory cytokine production associated with protection from subsequent infection and increased odds of symptoms once infected
Schofield *et al*. 2017^55^	*Pf*	Children in malaria‐endemic region	All γδ	Tim‐3 upregulated on γδ T cells following acute infection; frequency of Tim‐3^+^ γδ T cells higher among malaria‐exposed individuals compared to healthy controls	Individuals with asymptomatic malaria infection have higher proportions of Tim‐3^+^ γδ T cells
Taniguchi *et al*. 2017^56^	*Pf*	Adults and children with uncomplicated malaria	Non‐Vδ2	Non‐Vδ2 T cells expand during infection	Non‐Vδ2 T cells produce IL‐10 and IFNγ
Bediako *et al*. 2019^49^	*Pf*	Malaria‐exposed adults	All γδ	CD11c^+^ γδ T cells expanded in individuals with high numbers of malaria episodes and distinguished between high *vs*. low malaria episode groups	
Teirlinck *et al*. 2011^65^	Controlled human malaria infection (CHMI)^+^ chemoprophylaxis	Malaria‐naïve adults	All γδ	γδ T cells express effector memory phenotype	γδ T cells produce IFNγ even a year after infection
Seder *et al*. 2013^71^	Attenuated PfSPZ vaccination	Malaria‐naïve adults	All γδ	γδ T cells expanded following vaccination	Higher frequencies of γδ T cells correlate with protection after controlled human malaria infection
Ishizuka *et al*. 2016^69^	Attenuated PfSPZ vaccination	Malaria‐naïve adults	Vδ2^+^	γδ T cells expanded following immunisation	Higher frequencies of γδ T cells correlate with protection after controlled human malaria infection
Mordmuller *et al*. 2017^73^	Non‐irradiated PfSPZ vaccination + chemoprophylaxis	Malaria‐naïve adults	All γδ/Vγ9Vδ2	Dose‐dependent increase in the frequency of circulating γδ T cells (primarily the Vγ9Vδ2 subset)	Memory γδ T cells increase IFNγ secretion and expression of the activation marker CD38 post‐vaccination
Zaidi *et al*. 2017^72^	Irradiated PfSPZ vaccination	Malaria‐exposed adults	All γδ/Vδ2^+^	Vδ2^+^ T cells expanded following vaccination	Vδ2^+^ T cells significantly elevated among vaccinated individuals who remained uninfected during transmission season; number of memory Vδ2^+^ T cells associated with protection
Walk *et al*. 2019^66^	CHMI following BCG vaccination	Malaria‐naïve adults	All γδ	In half the BCG‐vaccinated individuals, CD69‐expressing γδ T cells expanded	Trends towards increased degranulation and granzyme B production among γδ T cells from BCG‐vaccinated volunteers compared to unvaccinated
Viral					
Fenoglio *et al*. 2011^96^	Influenza virus vaccination with MF59 adjuvant	HIV‐positive and HIV‐negative adults	Vδ1^+^	*In vivo* expansion of Vδ1^+^ γδ T cells in HIV+ individuals following vaccination	Expanded population produces anti‐fungal cytokines (may contribute to defence against opportunistic infections by compensating for impairment of CD4^+^ T cells)
Hoft *et al*. 2011^83^	Live attenuated influenza vaccine (LAIV) and inactivated influenza vaccine (TIV)	Children	All γδ	γδ T cells induced by LAIV, but not TIV	γδ T cells induced by vaccination with LAIV develop memory responses and inhibit viral replication
Horvath *et al*. 2012^85^	LAIV	Adult smokers and non‐smokers	All γδ	γδ T cells migrate to the lung following influenza infection in response to chemokines; cell population with characteristics of γδ T cells increases following LAIV vaccination	
Re *et al*. 2006^84^	Trivalent TIV	Elderly individuals	All γδ	Proliferative capacity of γδ T cells decreased and number of differentiated γδ T cells with effector/memory functions increased following vaccination	γδ T cells showed increased production of perforins after vaccination
Fausther‐Bovendo *et al*. 2008^89^	Human Immunodeficiency Virus (HIV)	HIV‐1‐infected adults	Vδ1^+^	Expansion of Vδ1^+^ T cells in individuals with HIV infection	Strong cytolytic capacities of Vδ1^+^ NKG2C^+^ T cells against HIV‐infected CD4 T cells
Garrido *et al*. 2018^100^	HIV	ART‐suppressed HIV‐infected adult men	All γδ	Vδ2^+^ T cells expanded up to 120‐fold in response to PAM/IL‐2 *ex vivo*	γδ T cells are capable of eliminating HIV‐infected targets and reduced viral replication up to 80%
He *et al*. 2013^98^	HIV	HIV‐positive and HIV‐negative adults	Vγ9Vδ2	CD16‐ and CD16^+^ Vδ2^+^ T‐cell subsets performed different functions in response to various stimuli	Potential for CD16^+^ Vδ2^+^ cells to control HIV infection via antibody‐dependent cell‐mediated cytotoxicity
Riedel *et al*. 2009^91^	HIV	HIV‐1‐infected adults that are natural viral suppressors (NVS)	Vγ9Vδ2	Depletion of Vγ9Vδ2 T cells occurs early in HIV disease; NVS patients demonstrated an increased number of Vγ9Vδ2 T cells	
Wallace *et al*. 1996^88^	HIV	Age not reported	All γδ	Increased numbers of γδ T cells in HIV‐1‐infected individuals	Anti‐HIV responses in a large proportion of Vγ9Vδ2 T cells may help explain the phenomenon of HIV exposure without infection
Worku *et al*. 2001^101^	Canarypox ALVAC‐HIV vCP205 and rgp120	Adults	All γδ	Induction of γδ T cells specific for canarypox (not HIV) antigens following vaccination	Expanded Vγ9^+^ γδ T cells produce IFNγ
Lafarge *et al*. 2001^106^	Cytomegalovirus (CMV)	Renal transplant patients	All γδ		Patients with γδ T‐cell expansion > 45 days after transplant had more severe symptoms than patients with early γδ T‐cell expansion; CMV infection resolves following γδ T‐cell expansion
Halary *et al*. 2005^108^	CMV	Renal‐ and lung‐transplanted patients with CMV	All γδ/Vδ2^−^	Vδ2^−^ T cells express receptors involved in intestinal homing	Numerous Vδ1^+^, Vδ3^+^ and Vδ5^+^ patient clones express TNFα, kill CMV‐infected targets and limit CMV growth *in vitro*; high frequency of these cells induce CD107a expression in the presence of CMV‐infected cells
Pitard *et al*. 2008^102^	CMV	Renal transplant patients with CMV and healthy adult donors (CMV seropositive/seronegative)	Vδ2^−^	Vδ2^−^ T cells expand and show effector/memory phenotype in transplanted patients and CMV+ healthy donors	Vδ2^−^ T cells from transplanted patients/CMV+ healthy donors show increased cytotoxicity in response to CMV *in vitro*; secondary response to CMV associated with a faster γδ T‐cell expansion and better resolution of infection compared to primary response
Knight *et al*. 2010^107^	CMV	Allogeneic stem cell transplant patients and healthy adult donors (CMV+/‐)	All γδ/Vδ2^−^	Long‐term expansion of Vδ2^−^ (not Vδ2^+^) T cells in transplant patients with CMV reactivation and in CMV+ healthy donors; restricted clonality	Vδ2^−^ T cells from CMV+ healthy donors and from a recipient of a graft from a CMV+ donor lysed CMV‐infected cells *in vitro*
Couzi *et al*. 2012^109^	CMV	Kidney transplant patients and healthy donors	All γδ/Vδ2^−^	High expression of CD16 on Vδ2^−^ T cells from CMV+ individuals	CD16^+^ γδ T cells did not mediate ADCC against CMV‐infected cells but produced IFNγ when incubated with IgG‐opsonised virions and inhibited CMV multiplication *in vitro*
Roux *et al*. 2013^104^	CMV	Adults from various age groups, pregnant women with primary infection, lung‐transplanted patients with primary or chronic infection	All γδ	CMV seropositivity leads to accumulation of highly differentiated Vδ2^−^ (but not Vδ2^+^) T cells; highest CD38 expression on γδ T cells from individuals with primary infection compared to chronic infection or no infection	
Alejenef *et al*. 2014^103^	CMV	Healthy adults and 2 immunocompromised individuals with symptomatic primary infection	Vδ2^−^	Highly differentiated effector memory Vδ2^−^ γδ T cells significantly increased in CMV+ healthy individuals compared to CMV‐ controls in all age groups	Vδ2^−^ T cells from CMV+ individuals contained higher levels of intracellular perforin and granzyme than CMV‐ individuals; Vδ2^−^ T cells do not immediately produce IFNγ/TNFα/CD107a following *ex vivo* incubation with CMV‐infected cells but do demonstrate effector functions after short‐term culture
Kallemeijn *et al*. 2017^113^	CMV	Healthy adults	All γδ	CMV associated with higher frequencies of γδ T cells with effector/memory and exhausted phenotypes	
Lee *et al*. 2017^105^	CMV	Renal transplant patients several years post‐transplant and healthy donors	All γδ/Vδ2^−^	Percentages of Vδ2^−^ T cells higher in CMV+ transplant patients and correlated with CMV antibody levels; Vδ2^−^ T cells skewed towards terminally differentiated phenotype; many Vδ2^−^ T cells in CMV+ individuals express CD8	Expression of CD107a and production of IFNγ by Vδ2^+^ and Vδ2− γδ T cells in response to staphylococcal enterotoxin B was not altered by CMV

To maximise functional responses in future similar studies, it will be important to improve our understanding of the timing of γδ T‐cell *vs*. αβ T‐cell responses following vaccination, as well as any potential negative effects of overstimulation of γδ T cells. As specific subsets of γδ T cells that correlate with protection in different contexts are identified, optimisation of methods to specifically target these subsets will be beneficial. Especially given the hypothetical possibility of γδ T‐cell anergy/exhaustion, it will be essential to define responses that optimally stimulate and antigens/agonists that best elicit that response. Altogether, as development of vaccines targeting infectious diseases that have long proved elusive becomes more of a reality, it will be important to broaden our perspective beyond targeting antibody‐driven or T‐cell responses and to intentionally target innate cells, such as γδ T cells.

## Conflict of interest

The authors declare no conflict of interest.
